# Supplementary material for: Complexome profiling of the Chlamydomonas psb28 mutant reveals TEF5 as an early PSII assembly factor
Source: Plant Cell. 2025 Mar 17;37(6):koaf055. doi: 10.1093/plcell/koaf055 (PMC12164590; doi:10.1093/plcell/koaf055)
Supplement: koaf055_Supplementary_Data [file koaf055_supplementary_data.zip › psb28_interactive.zip!/plots/intensity/qualityPlots/peptides/AOT3_Cre03.g168550.t1.1.html]

### Cre03.g168550.t1.1

Trivial name: AOT3  
SeqCoverage [median]: 13.30  
Mass: 48.57 kDa  
  
 

| Sequence | MissedCleavage | Mass [Da] | wtIntensity | muIntensity | Score |
| --- | --- | --- | --- | --- | --- |
| AAATAAGAPA | 0 | 770.39 | 1702.30 | 4460.48 | 134.06 |
| FALTMEPPAAALQGVIPGAK | 0 | 1981.07 | 5782.17 | 0.00 | 121.27 |
| TALAIGILLAAR | 0 | 1181.75 | 437.49 | 0.00 | 78.33 |
| TGGGPVGYEEIAEAAFGPLGR | 0 | 2047.00 | 3323.51 | 0.00 | 175.28 |
|  |  |  | 11245.5 | 4460.5 |  |
